# Efficacy and safety of esketamine for sedation during colonoscopy: A systematic review and Meta-analysis of randomized controlled trials

**DOI:** 10.1007/s00228-025-03935-2

**Published:** 2026-01-17

**Authors:** Zainab Hussein, Amira Mohamed Taha, Alaa Abdrabou Abouelmagd, Mohamed Nasser Elshabrawi, Abdul Karim Durvesh, Eman Ayman Nada, Mohamed Abuelazm, Mohamed Elnaggar, Ismail Elkhattib

**Affiliations:** 1 Faculty of Medicine, Minia University, Minia, Egypt; 2 Faculty of Medicine, Fayoum University, Fayoum, Egypt; 3 Faculty of Medicine, South Valley University, Qena, Egypt; 4 Clinical Research Department, Aswan Heart Center, Magdi Yaqoup Foundation, Aswan, Egypt; 5 Sindh Institute of Advanced Endoscopy and Gastroenterology (SIAG), Karachi, Pakistan; 6 Faculty of Pharmacy, Tanta University, Gharbia, Egypt; 7 Ministry of health, Damietta, Egypt; 8 Faculty of Medicine, Tanta University, Tanta, Egypt; 9 Hartford Healthcare, Hartford, CT USA; 10 University of Nebraska Medical Centre, Omaha, NE USA

**Keywords:** Endoscopy, Propofol, Esketamine, Colonoscopy sedation, Anaesthesia, Endoscope

## Abstract

**Background:**

Hemodynamic instability during colonoscopy sedation remains a significant clinical concern. Esketamine’s sympathomimetic properties may protect against these risks while reducing sedative requirements. Hence, we aim to evaluate the efficacy and safety of esketamine in improving intraprocedural sedation during colonoscopy.

**Methods:**

We systematically searched PubMed, Scopus, CENTRAL, and Web of Science until June 2025 for randomized controlled trials. The primary outcome was the incidence of intraprocedural hypotension; secondary outcomes included bradycardia, hypoxemia, and recovery parameters. Dichotomous outcomes were pooled using risk ratios (RR) and continuous outcomes using standardized mean differences (SMD), with heterogeneity assessed via I² statistics. PROSPERO ID: CRD420251105691.

**Results:**

Five randomized controlled trials comprising 858 patients were included in our analysis. Esketamine significantly reduced the risk of intraprocedural hypotension (RR: 0.34, 95% CI 0.22–0.53; I²=58%) and the incidence of hypoxemia (RR: 0.38, 95% CI 0.19–0.73; I²=0%). A reduction in injection pain was also observed (RR: 0.42, 95% CI 0.19–0.97; I²=80.5%), though this finding showed sensitivity in leave-one-out analysis. No significant differences were found between groups in bradycardia risk (RR: 0.51, 95% CI 0.23–1.14), total propofol requirement (SMD: -0.23, 95% CI -0.50 to 0.04), induction time, or procedure duration. The reduction in hypotension remained robust in sensitivity analyses.

**Conclusion:**

Esketamine significantly enhanced hemodynamic stability and reduced sedative demand during colonoscopy without delaying recovery, supporting its use in high-risk patients.

**Supplementary Information:**

The online version contains supplementary material available at 10.1007/s00228-025-03935-2.

## Introduction

Colonoscopy diagnoses and treats colorectal diseases such as polyps, tumors, and inflammation. It also serves as a primary screening test for colorectal cancer [1]. During a regular colonoscopy, patients may have elevated blood pressure, tachycardia, and restlessness due to tension, abdominal distension, and pain stimulation, which raises the risk of adverse effects [2]. A painless colonoscopy reduces patient anxiety, improves endoscopists’ operating circumstances, and enhances positive patient detection and review rates [3].

Visual or audiovisual distraction was found in recent evidence to ease pain and anxiety during colonoscopy and improve patient satisfaction [4]. Based on recent data, abdominal compression devices may also shorten intubation time, lessen discomfort, and reduce the need for postural changes [5]. Regarding polypectomy techniques, cold snare resection is associated with shorter procedure times and fewer adverse events, while hot snare offers higher complete resection rates [6].

Using sedatives and analgesics to induce slight central nervous system depression is known as conscious sedation. Colonoscopy procedures are better performed and go more easily when patients can change their position as instructed by the endoscopist and remain receptive to verbal cues [7]. Patients who are at risk of problems from deeper sedation levels will benefit most from this strategy [8]. Propofol is commonly used for anesthetic sedation; however, it can increase the risk of cardiovascular and respiratory depression in a dose-dependent way [9]. Fentanyl has been widely used for deep sedation during painful procedures due to its analgesic features [10, 11]. This typical regimen is frequently prescribed for patients undergoing endoscopic resection. However, this combination causes hemodynamic instability and respiratory depression, leading to adverse consequences in clinical settings. A medication that reduces propofol dosage without causing side effects should improve clinical safety, especially for patients with advanced age or comorbidities, who possess high-risk factors for propofol-related adverse events.

Esketamine is the S-enantiomer of ketamine and an N-methyl-D-aspartate (NMDA) receptor antagonist [12, 13]. It has more potent sedative and analgesic effects than racemic ketamine [12, 13]. Additionally, it has a lower frequency of adverse consequences and positive sympathomimetic effects [14, 15]. Esketamine is ideal for rapid and painless outpatient diagnostics because of its quick onset and short elimination duration. Additionally, by raising heart rate, relaxing bronchial smooth muscle, and lowering airway resistance, its sympathomimetic activity reverses the respiratory and circulatory depression brought on by propofol [16, 17]. The safety and efficacy of esketamine and propofol conscious sedation in colonoscopies have not been thoroughly investigated, and the currently available data indicate conflicting results. Thus, our study aimed to investigate the safety and effectiveness of esketamine and propofol when administered together for conscious sedation during elective colonoscopy.

## Methodology

### Protocol and registration

This systematic review and meta-analysis was conducted in accordance with the Preferred Reporting Items for Systematic Reviews and Meta-Analyses (PRISMA) guidelines [18]. The study protocol was prospectively registered in the International Prospective Register of Systematic Reviews (PROSPERO) under the registration number [CRD420251105691].

### Eligibility criteria

Randomized controlled trials (RCTs) were eligible if they compared esketamine, administered as an adjunct to propofol-based sedation, with control regimens (propofol alone or propofol combined with an opioid) in adult patients undergoing elective colonoscopy. Studies were required to report at least one predefined primary or secondary outcome. The primary outcomes included the incidence of intraprocedural hypotension, as defined by each included study, and total sedative requirements. Also, secondary outcomes included the incidence of bradycardia, hypoxemia, injection pain, total propofol dose, induction time, recovery time, and procedure duration. Trials enrolling pediatric patients, non-colonoscopy procedures, or administering racemic ketamine for sedation without esketamine-specific data were excluded.

### Data sources and study selection

A comprehensive search was conducted in PubMed, Scopus, the Cochrane Central Register of Controlled Trials (CENTRAL), and Web of Science, without language restrictions, from inception to June 2025. Detailed search strategies are provided in (Table S1). Reference lists of included trials and relevant reviews were manually screened to identify additional eligible studies. Two reviewers, M.N.E. and E.A.N., independently screened records and assessed full-text articles, resolving disagreements by a third reviewer. Any study that did not meet the inclusion criteria was excluded, and the reason for exclusion was recorded.

### Data extraction

Using a standardized pilot-tested extraction form, three authors, M.N.E., A.D., and E.A.N., extracted data independently, and to ensure accuracy and consistency, the extraction process was repeated in duplicate. Disagreements were resolved by discussion among the reviewers. Extracted information included study identifiers, country, study design, sample size with group allocation, participant demographics, ASA classification, comorbidities, baseline vital signs (heart rate, systolic and diastolic blood pressure, and peripheral oxygen saturation), intervention and comparator regimens (drug, dose, route, timing), sedation protocols, and results.

Outcome data were extracted for all pre-specified primary and secondary outcomes. For dichotomous outcomes (e.g., incidence of hypotension, bradycardia), the number of events and the total number of patients assessed in each group were extracted. For continuous outcomes (e.g., total propofol dose, induction time), each group’s mean, standard deviation (SD), and sample size were collected. When continuous outcomes were reported using median, interquartile range (IQR), or range, the sample mean and standard deviation were estimated using the validated methods described by Wan et al. [19] to permit inclusion in the meta-analysis.

### Risk of bias assessment

Risk of bias was assessed using the Cochrane Risk of Bias 2 (ROB-2) [20] tool across five domains: (D1) bias arising from the randomization process, (D2) bias due to deviations from intended interventions, (D3) bias due to missing outcome data, (D4) bias in measurement of the outcome, and (D5) bias in selection of the reported result. Based on the domain-level judgments, an overall risk of bias was assigned for each study, categorized as “low risk,” “some concerns,” or “high risk.” Three reviewers, M.N.E., A.D., and E.A.N., independently performed assessments, with disagreements resolved through consensus. ROB-2 findings are visualized in a traffic light plot.

### Statistical analysis

Statistical analysis was conducted using R (version 4.4.2) [21], R Studio [22], and the meta package [23]. Forest plots were generated using risk ratio (RR) for dichotomous outcomes and standardized mean difference (SMD) for continuous outcomes, each of which was presented with its respective 95% confidence intervals (CI). A random-effect model was adopted rather than a fixed-effect model, yielding a more conservative estimate of the pooled effect and generalizable results. Significant heterogeneity was specified as a P-value less than 0.1 or a Higgins and Thompson I-squared value exceeding 50%. Assessment of publication bias via visual assessment of funnel plots was not possible due to the inadequate number of the included studies. A p-value less than 0.05 was considered statistically significant. Finally, leave-one-out sensitivity analysis was conducted by excluding one study at a time (leave-one-out method) to investigate each study’s influence on the overall effect size estimate.

## Results

### Study selection process

**Fig. 1:**
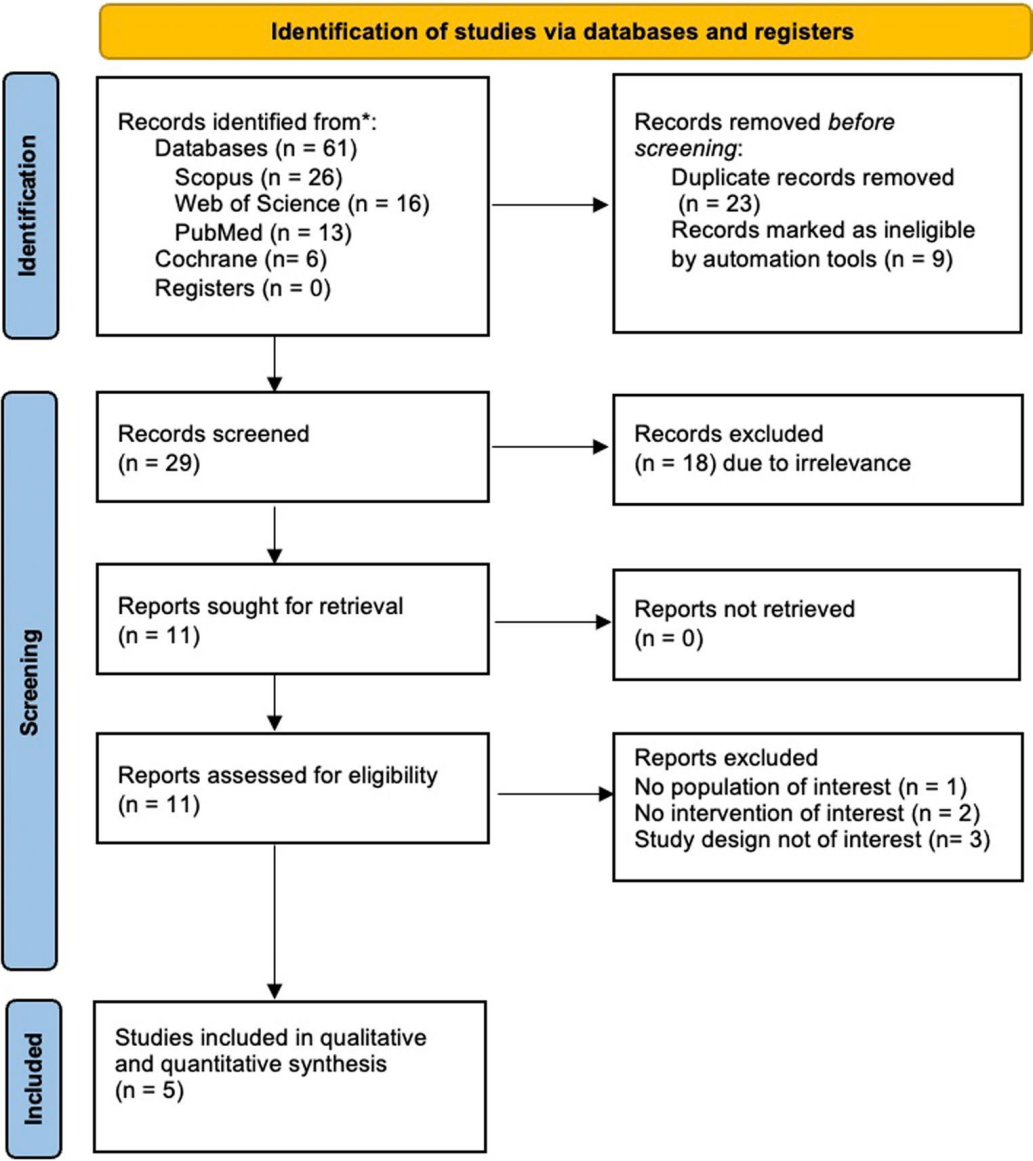
PRISMA flow chart of the screening process

We identified 61 unique records through database searches and manual screening. After removing duplicates, we screened 44 records by title and abstract. Eleven full-text articles underwent eligibility assessment, and we finally included five RCTs [24–28]. The PRISMA flow diagram of the selection process is presented in Fig. 1.

### Study characteristics

All included RCTs were conducted in China and enrolled adult patients undergoing elective colonoscopy. Sample sizes ranged from 100 to 252 participants, with a total of 423 patients. Esketamine was administered intravenously as an adjunct to propofol at doses ranging from 0.15 to 0.4 mg/kg. Comparator regimens consisted of propofol alone or propofol combined with an opioid. Sedation protocols, patient monitoring methods, and outcome definitions were largely consistent across studies. Key study characteristics are summarized in Table 1, and baseline demographic and clinical characteristics are presented in Table 2.

**Table 1:** Summary characteristics of the included RCTs

Study ID	Country	Design	Population/Setting	Intervention	Comparator	*N* total (*N*/group)	Primary Outcome	Secondary Outcomes
Ma et al., 2024	China	Randomized Controlled Trial (Double-blind)	Adults undergoing painless curative colorectal endoscopic resection	Esketamine 0.25 mg/kg + Propofol 1.5 mg/kg IV bolus	Propofol 1.5 mg/kg IV bolus	160 (81/79)	Total propofol dose	Sedation depth (MOAA/S), recovery time (Aldrete), patient & endoscopist satisfaction, adverse events
Sun et al. 2024	China	Randomized Controlled Trial (Double-blind)	Adults undergoing painless colonoscopy	Esketamine 0.15 mg/kg + Propofol 1.5–2.0.5.0 mg/kg + Maintenance: Propofol 0.3–0.5 mg/kg	Sufentanil 0.1 µg/kg + Propofol 1.5–2.0.5.0 mg/kg + Maintenance: Propofol 0.3–0.5 mg/kg	151 (76/75)	Postoperative fatigue	ICFS-10 scores at baseline and 1 day post-colonoscopy, time to discharge and patients’ satisfaction.
Fu et al. 2024	China	Randomized Controlled Trial (4-arm)	Adults undergoing colonoscopy under propofol-based sedation	propofol 1 mg/kg + Esketamine 0.2 mg/kg (E1), 0.3 mg/kg (E2), 0.4 mg/kg (E3)	propofol 2 mg/ kg	100 (25/25/25/25)	Incidence of hypotension	cardiovascular side effects other than hypotension, incidence of hypoxia, cumulative changes in cardiovascular and respiratory parameters, total propofol dosage, anesthesia recovery time, and satisfactory levels
Xiao et al. 2024	China	Randomized Controlled Trial (Double-blind, 3-arm)	Adults undergoing painless colonoscopy	CS2: Esketamine 0.2 mg/kg + Propofol 0.5–0.8 mg/kg + Maintenance: Propofol 0.3 mg/kg	DS: Placebo + Propofol 1.5–2.0.5.0 mg/kg + Maintenance: Propofol 0.3 mg/kg, CS1: Sufentanil 0.1 µg/kg + Propofol 0.5–0.8 mg/kg + Maintenance: Propofol 0.3 mg/kg	195 (65/65/65)	The incidence of hypoxemia, hypotension, hypertension, and bradycardia and excellent and good rates of anaesthesia	perioperative changes in vital signs (MAP, HR, and SpO2), anaesthesia induction time, dischargeable time, satisfaction scores, and incidence of postoperative nausea and vomiting (PONV), drowsiness, dizziness, propofol injection pain, assisted ventilation and vasoactive medications.
Liu et al. 2025	China	Randomized Controlled Trial (Double-blind)	Adults undergoing outpatient colonoscopy	Esketamine 0.2 mg/kg IV + propofol 1 mg/kg + Maintenance: Propofol: 0.5 mg/kg IV bolus	Placebo IV	252 (126/126)	Cognitive recovery on postoperative day 3	Overall recovery, recovery in other PostopQRS domains, time to discharge, and adverse events.

*IV = intravenous; MOAA/S = Modified Observer’s Assessment of Alertness/Sedation scale; ICFS-10 = 10-item Identity-Consequence Fatigue Scale; CS = combination sedation; DS = drug sedation; MAP = mean arterial pressure; HR = heart rate; SpO₂ = peripheral capillary oxygen saturation; PONV = postoperative nausea and vomiting; PostopQRS = Postoperative Quality of Recovery Scale.*

**Table 2:** Baseline characteristics of the participants

Study ID	Age (years)	Sex (M/F)	BMI (kg/m²)	ASA status	Baseline HR (bpm)	Baseline SBP (mmHg)	Baseline DBP (mmHg)	SpO2(%)
Ma et al. 2024	E: 61.0 ± 11.1; Fentanyl: 60.5 ± 10.8	Esketamine: 49/17; Fentanyl: 48/31	Esketamine: 23.9 ± 3.1; Fentanyl: 23.2 ± 3.5	I-III	Esketamine: 73.6 ± 11.3; Fentanyl: 73.9 ± 10.8	Esketamine: 132.7 ± 17.6; Fentanyl: 134.8 ± 15.3	Esketamine: 73.9 ± 10.8; Fentanyl: 78.7 ± 11.1	Esketamine: 99.3 ± 1.2; Fentanyl:99.3 ± 0.9
Sun et al. 2024	Esketamine: 44.46 ± 11.92; Sufentanil:46.03 ± 12.91	Esketamine: 35/41; Sufentanil: 36/39	Esketamine: 23.24 ± 2.84; Sufentanil: 23.63 ± 2.75	I-II	NR	NR	NR	NR
Fu et al. 2024	Group P: 54.4 ± 2.7; Group E1: 49.88 ± 2.6; Group E2: 48.9 ± 2.4; Group E3: 48.5 ± 3.0	P: 10/15; E1: 11/14; E2: 12/13; E3: 16/9	P: 24.5 ± 0.9; E1:23.8 ± 0.7; E2: 24.0 ± 0.6; E3: 23.4 ± 0.6	I-II	P: 76.9 ± 2.8; E1: 74.1 ± 2.5; E2: 76.8 ± 2.0; E3: 72.7 ± 2.0	P: 129.12 ± 21.58; E1: 127.04 ± 23.43; E2: 126.40 ± 19.36; E3: 75.56 ± 17.64	P: 78.52 ± 16.35; E1: 78.76 ± 13.58; E2: 78.12 ± 13.41; E3: 120.84 ± 20.11	P: 99.76 ± 0.52; E199.56 ± 1.04; E2: 99.44 ± 1.08; E3: 99.56 ± 1.50
Xiao et al. 2024	CS2: 48.9 ± 11.8; DS: 47.7 ± 11.4; CS1: 48.2 ± 11.5	CS2: 25/40; DS: 30/35; CS1: 29/36	CS2: 22.8 ± 1.8; DS: 22.3 ± 2.1; CS1: 22.0 ± 2.3	I-II	NR	NR	NR	NR
Liu et al. 2025	Esketamine: 64.5 [54–69]; Placebo: 65 [55–69]	Esketamine: 69/57; Placebo: 64/65	Esketamine: 22.7 [21.5–24.4]; Placebo: 23 [21.8–24.3]	I-III	NR	NR	NR	NR

BMI = body mass index; ASA = American Society of Anesthesiologists physical status classification; HR = heart rate; bpm = beats per minute; SBP = systolic blood pressure; DBP = diastolic blood pressure; SpO₂ = peripheral capillary oxygen saturation; NR = Not reported in the primary study or insufficient information provided

### Risk of bias assessment

**Fig. 2:**
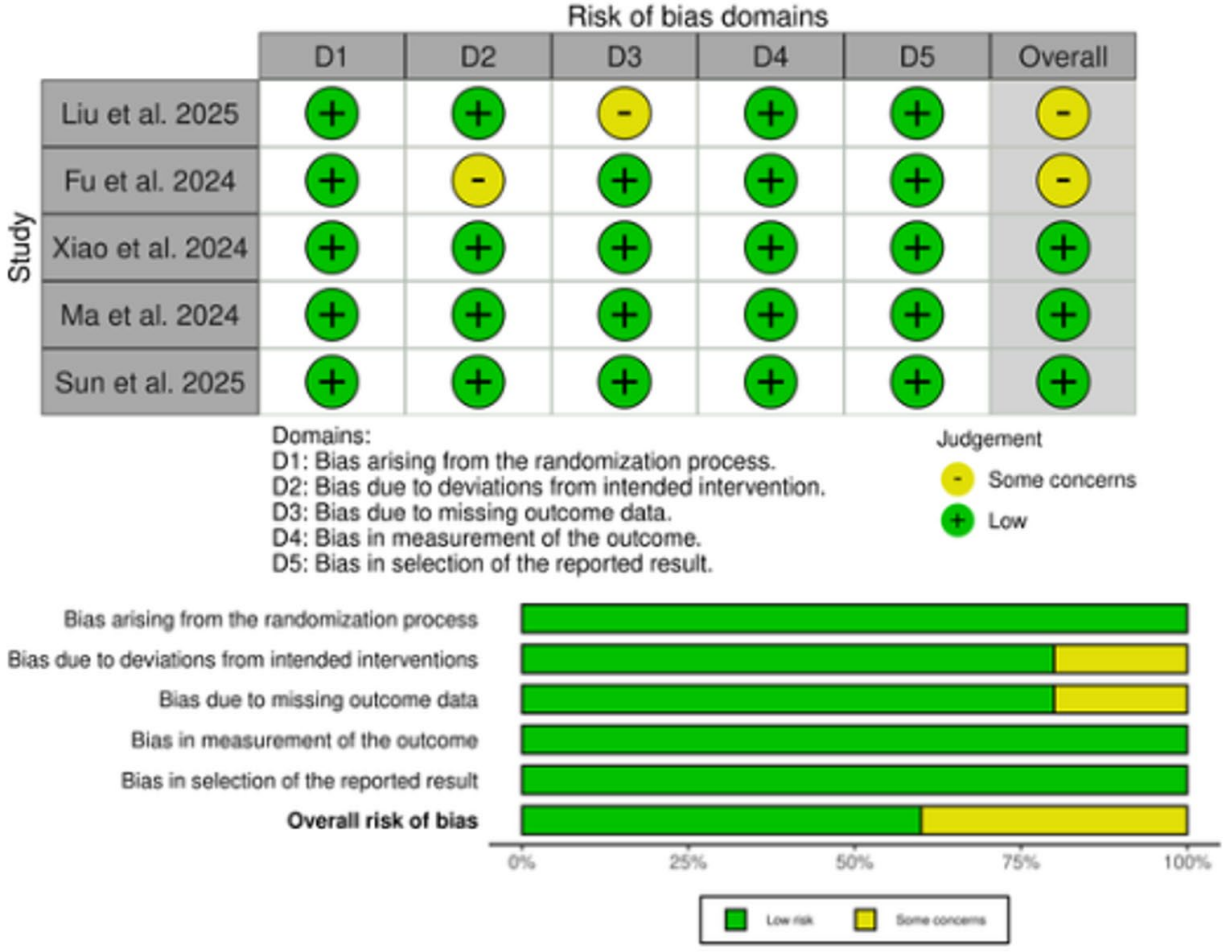
Quality assessment of risk of bias in the included trials. The upper panel presents a schematic representation of risks (low = green, unclear = yellow, and high = red) for specific types of biases of the studies in the review. The lower panel presents risks (low = red, unclear = yellow, and high = red) for the subtypes of biases of the combination of studies included in this review

Risk of bias assessment indicated that all five trials were at low risk of bias for most domains, with only one trial having minor concerns in allocation concealment. Overall, three studies were rated as “low risk” and two as having “some concerns,” primarily due to missing data or incomplete reporting of blinding procedures **(**Fig. 2**)**.

### Primary outcomes: intraprocedural hypotension

**Fig. 3:**
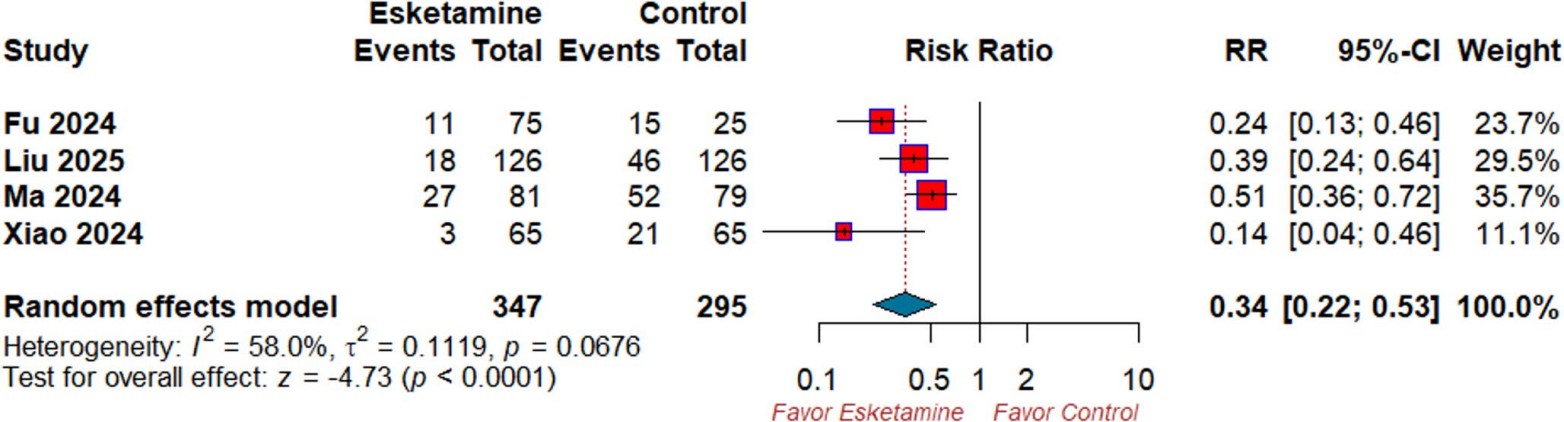
Forest plot of intraprocedural hypotension. RR: risk ratio, CI: confidence interval

Pooled analysis demonstrated that esketamine-based sedation regimens significantly reduced the risk of hypotension (RR 0.34; 95% CI 0.22–0.53; *p* < 0.0001; I² = 58%) **(**Fig. 3**)**. After the sensitivity analysis, the significant heterogeneity was best resolved after excluding Ma et al. 2024 (I^2^ = 35.9%) (Table S2).

### Secondary outcomes

**Fig. 4:**
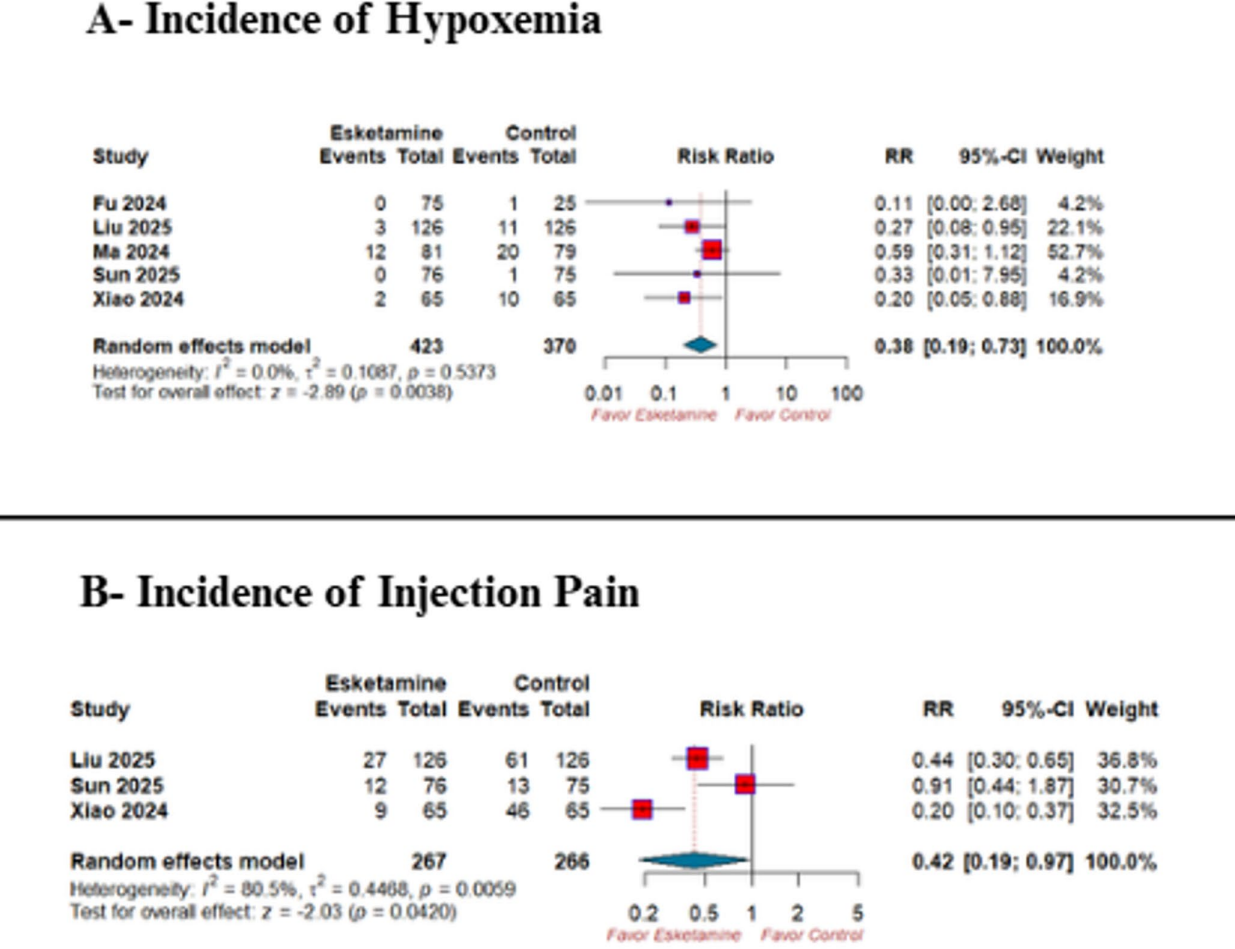
Forest plot of the key secondary outcomes: **(A)** hypoxemia and **(B)** injection pain

**Fig. 5:**
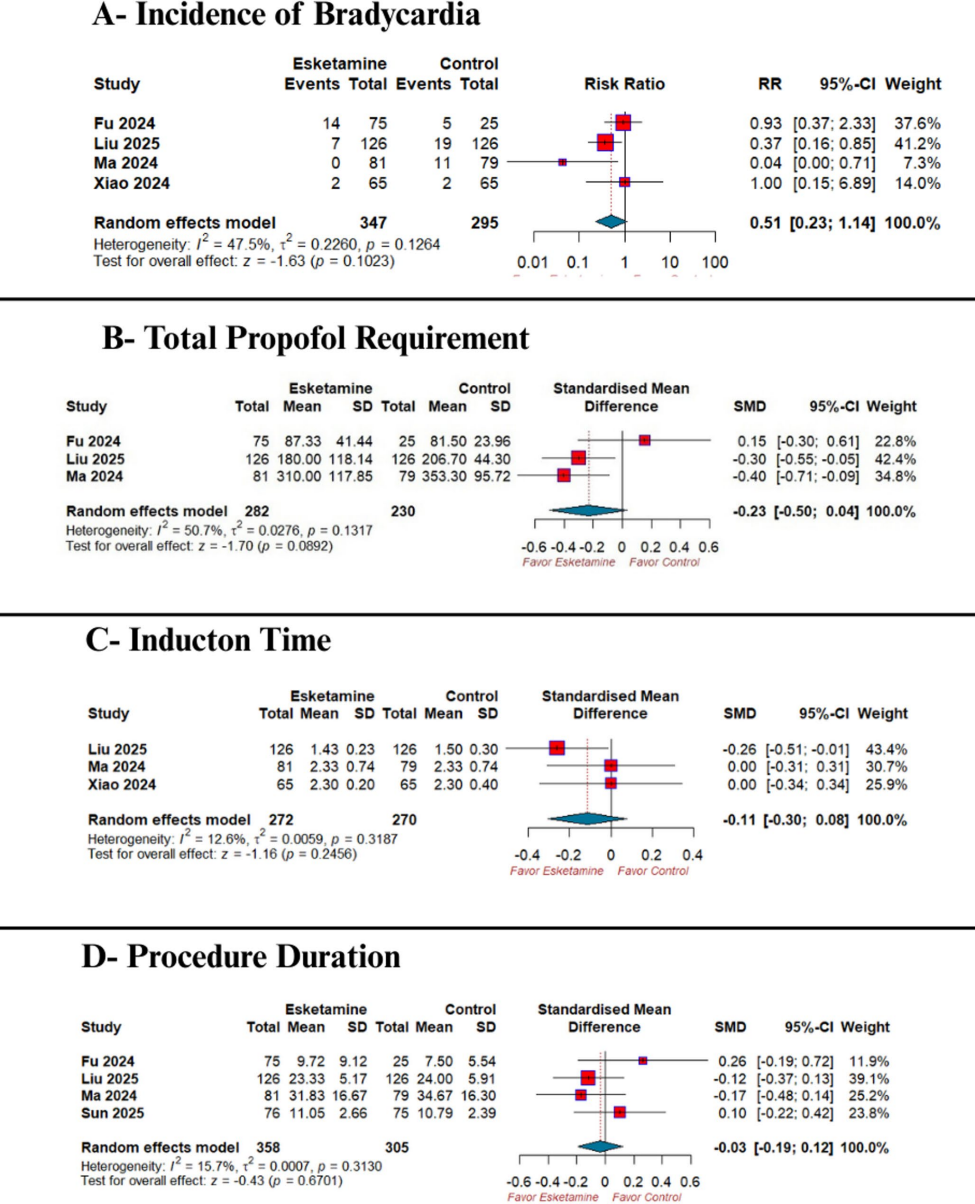
Forest plot of other secondary outcomes: **(A)** bradycardia, **(B)** total propofol requirement, **(C)** induction time, and **(D)** procedure duration

Esketamine significantly decreased the risk of hypoxemia (RR 0.38; 95% CI 0.19–0.73; *p* = 0.0038; I² = 0.0%) **(**Fig. 4-A), and the incidence of injection pain (RR 0.42; 95% CI 0.19–0.97; *p* = 0.04; I² = 80.5%) **(**Fig. 4-B**)** compared with the control group. However, There was no significant difference between both groups regarding the risk of bradycardia (RR 0.51; 95% CI 0.23–1.14; *p* = 0.10; I² = 47.5%) **(**Fig. 5-A**)**, total propofol requirement (SMD − 0.23; 95% CI −0.50 to 0.04; *p* = 0.09; I² = 50.7%) **(**Fig. 5-B), induction time (SMD − 0.11; 95% CI − 0.30 to 0.08; *p* = 0.24; I² = 12.6%) **(**Fig. 5-C**)**, or procedure duration (SMD − 0.03; 95% CI − 0.19 to 0.12; *p* = 0.67; I² = 15.7%) **(**Fig. 5-D**).**

### Sensitivity analysis

Leave-one-out sensitivity analyses were performed for outcomes with substantial heterogeneity (I² > 50%), including hypotension (I² = 58%), injection pain (I² = 80.5%), and propofol requirement (I² = 50.7%). Complete results are provided in (Table S2). The significant treatment effect for hypotension (RR 0.34, 95% CI 0.22–0.53) remained stable throughout sensitivity testing. All recalculated estimates maintained statistical significance (*p* < 0.01) with consistent effect sizes (RR range: 0.28–0.39).

Injection pain results (RR 0.42, 95% CI 0.19–0.97) demonstrated volatility. Exclusion of any individual study eliminated statistical significance while producing considerable variation in point estimates (RR range: 0.31–0.59). Also, propofol requirement analysis revealed particular sensitivity to the exclusion of Fu et al. [26]. Their removal altered the significance (SMD − 0.34, 95% CI −0.53 to −0.19; *p* = 0.0007) and heterogeneity (I² = 0%).

## Discussion

In this meta-analysis, we included five recent RCTs that assessed the safety and efficacy of esketamine and propofol sedation in patients with elective colonoscopy. When compared to control regimens, esketamine significantly decreased the risk of intraprocedural hypotension. Esketamine also reduced the incidence of bradycardia and hypoxemia, though the difference was not statistically significant. Esketamine administration resulted in a moderate reduction in the risk of injection pain, but had no meaningful effect on propofol dose requirements, induction time, or procedure duration.

Numerous studies have shown that the combination of propofol and esketamine lowers the necessary propofol dosage when compared to propofol-fentanyl regimens. That esketamine can counteract the cardiopulmonary depressive effects of propofol [24–29]. Studies consistently reported the advantage of esketamine in reducing the recurrence of hypotension and bradycardia, supporting our findings [24–28]. Liu et al. reported better hemodynamic results with esketamine adjunct therapy, such as decreased rates of bradycardia, hypotensive episodes, oxygen desaturation, and local injection pain [24]. These clinical benefits did not increase the likelihood of psychotomimetic problems, lengthen recovery, or post-procedural discharge time [24]. The esketamine cohort showed consistently superior patient satisfaction scores [24].

The decrease in propofol consumption and the intrinsic sympathomimetic effects of esketamine might be responsible for this improved hemodynamic stability [30, 31]. These actions seem to work together to adequately counteract the respiratory and circulatory depression brought on by propofol. According to available evidence, esketamine has been reported to cause increases in both systolic and diastolic blood pressure, focusing on the rise of systolic blood pressure [32]. This result supports the hypothesis that an underlying mechanism could be elevated cardiac output [33]. Nevertheless, esketamine administration causes a transient rise in blood pressure that rarely results in major cardiovascular events [34]. In contrast to earlier findings of increased tachycardia rates with esketamine, Ma et al. found no consistent increase in tachycardia incidence, even though bradycardia was less frequent with esketamine than with fentanyl [35]. Higher dosages of propofol or the recognized bradycardic effects of fentanyl through cardiovascular depression could be the cause of this finding [36].

Our findings demonstrated a significant reduction in the incidence of hypoxemia in the esketamine group. Recent evidence from a rigorous multicenter clinical trial indicates that incorporating 0.15 mg/kg esketamine into propofol-based sedation protocols substantially decreases the occurrence of combined hypotensive and oxygen desaturation episodes during gastrointestinal endoscopic procedures [39]. Complementary findings by Eberl et al. revealed that this exact esketamine dosage (0.15 mg/kg) effectively decreased total propofol requirements during endoscopic retrograde cholangiopancreatography while maintaining comparable recovery durations, patient and endoscopist satisfaction levels, and safety profiles relative to alfentanil-based regimens [29]. Notably, the esketamine protocol showed no increase in procedure-related complications or cardiorespiratory adverse events [29].

According to Song et al., low-dose esketamine reduced the incidence of desaturation and hypotension during endoscopy by about 61% [39]. However, there was no change in respiratory events reported by Ma et al., even with a lower dosage of propofol and the respiratory stimulatory effects of low-dose esketamine [27, 31]. This may be because most patients had Mallampati scores ≤ 2, and the dosage reduction might not have been enough to lower the frequency of these instances. Additionally, respiratory depression is less common when using the propofol target-controlled infusion [40].

Fu et al. found no significant difference in the occurrence of hypoxia and respiratory parameters [26]. Esketamine’s effect on respiration is unknown and may vary by dose. Esketamine has been shown to increase respiration at sub-anesthetic dosages [38], whereas greater levels may decrease breathing [42]. Fu et al. reported that they did not deliver opioids because the respiratory depression caused by propofol is often temporary. Propofol’s effect on respiratory measurements varies based on dose and age [43, 44]. The authors indicated the low propofol dose, resulting in minor respiratory effects. Finally, unlike gastroscopy, colonoscopy does not need airway intubation and has less impact on breathing.

According to a recent meta-analysis, individuals undergoing endoscopy may benefit from a low dosage of esketamine (0.15 mg/kg) and propofol [37]. Nevertheless, 0.25 mg/kg esketamine provided extra advantages regarding mean arterial pressure, involuntary movements, hypoxemia, respiratory depression, and recovery and induction time [37]. Among the included studies, Sun et al. used a dose of 0.15 mg/kg of esketamine for their investigation based on these results. A nationwide survey in China reported that the combination of low-dose sufentanil and propofol is the regular sedation method that should be used in endoscopy [38]. Hence, the authors compared esketamine or sufentanil before anesthesia induction. According to their study [28], there were no appreciable variations in postoperative pain or propofol dosage between the groups, suggesting that 0.15 mg/kg esketamine functions similarly as an adjuvant analgesic to 0.1 µg/kg sufentanil.

In our analysis, there were no notable intergroup differences in procedure duration, induction time, or recovery time, which is in line with research showing similar findings for opioid and esketamine [24–28]. Notably, evidence indicates that older patients receiving esketamine-propofol combinations recover rapidly [45], which may be because these patients require less propofol.

### Strengths and Limitations

To our knowledge, this is the most thorough and recent meta-analysis assessing the efficacy and safety of low-dose esketamine as an adjunct to propofol sedation for colonoscopy procedures. We restricted our inclusion criteria to RCTs that offered the best quality evidence supporting the validity of results. In addition, we adhered strictly to the PRISMA Checklist and performed sensitivity analyses to evaluate the robustness of the findings and consider study heterogeneity. However, there are several limitations. The small number of included studies and the limited sample size may reduce the statistical power of our analysis. Furthermore, all included studies were conducted in China, which may affect the generalizability of the results to other populations. The baseline characteristics of the Chinese population, such as a predominance of patients with certain health profiles (e.g., ASA grades I-II), may differ from those in other regions, where the patient demographic could be more varied. Also, some of the reported results were heterogeneous. These differences could influence the broad applicability of the results to populations with different demographic profiles.

### Implications for Future Research

Our findings support using esketamine as an adjunct to propofol sedation for colonoscopy procedures. However, heterogeneity was present among some of our reported outcomes; hence, caution should be exercised while interpreting our findings. Larger, multicenter trials are necessary to validate these findings across multiple patient demographics and healthcare settings. Consistent outcome definitions should be utilized in similar investigations to eliminate any potential biases or variability observed in the current study.

## Conclusion

Esketamine, when administered with propofol, considerably improves hemodynamic stability and decreases propofol requirements during colonoscopy while not extending induction or recovery times. However, regional constraints, heterogeneity in some outcomes, and limited sample size highlight the importance of multicenter RCTs to validate these findings in different populations.

## Supplementary Information

Below is the link to the electronic supplementary material.

Supplementary Material 1 (25.5 KB)

## Data Availability

Not applicable.

## References

[1] Chen C, Stock C, Hoffmeister M, Brenner H (2019) Optimal age for screening colonoscopy: a modeling study. Gastrointest Endosc 89(5):1017–1025 30639539 10.1016/j.gie.2018.12.021

[2] Sun DJ, You YX, He XJ, Li HT, Zeng XP, Li DZ et al (2022) Effects of light music played by piano intervention on satisfaction, anxiety, and pain in patients undergoing colonoscopy: a randomized controlled trial. Medicine 101(52):e32339 36595974 10.1097/MD.0000000000032339 PMC9803447

[3] Shi CM, Zhou Y, Yang N, Li ZQ, Tao YF, Deng Y et al (2023) Quality of psychomotility recovery after Propofol sedation for painless gastroscopy and colonoscopy. Beijing Da Xue Xue Bao 55(2):324–327 37042144 10.19723/j.issn.1671-167X.2023.02.017 PMC10091250

[4] Saab O, Al-Obaidi H, Merza N, Bhagat U, Al-Sagban A, Algodi M et al (2024) The impact of visual distraction interventions on patients’ pain and anxiety during colonoscopy: a systematic review and meta-analysis of randomized controlled trials. J Clin Gastroenterol 58(3):235–244 10.1097/MCG.0000000000002086 39495815

[5] Abuelazm M, Awad AK, Mohamed I, Mahmoud A, Shaikhkhalil H, Shaheen N et al (2023) The impact of abdominal compression devices on colonoscopy outcomes: a systematic review and meta-analysis of randomized controlled trials. Curr Med Res Opin 39(10):1329–1339 37526014 10.1080/03007995.2023.2243214

[6] Abuelazm MM, Mohamed I, Jaber FS, Katamesh BE, Shaikhkhalil H, Elzeftawy MA et al (2023) Cold versus hot snare polypectomy for colorectal polyps: an updated systematic review and meta-analysis of randomized controlled trials. J Clin Gastroenterol 57(8):760–73 36787428 10.1097/MCG.0000000000001837

[7] Kang S, Lu J, Zhou HM (2021) Anesthetic strategy for obese patients during gastroscopy: deep sedation or conscious sedation? A prospective randomized controlled trial. J Anesth 35(4):555–562 34052943 10.1007/s00540-021-02951-7

[8] Stefanutto TB, Feiner J, Krombach J, Brown R, Caldwell JE (2012) Hemoglobin desaturation after propofol/remifentanil-induced apnea: a study of the recovery of spontaneous ventilation in healthy volunteers. Anesth Analg 114(5):980–986 22492188 10.1213/ANE.0b013e31824e5bc4

[9] Wadhwa V, Issa D, Garg S, Lopez R, Sanaka MR, Vargo JJ (2017) Similar risk of cardiopulmonary adverse events between Propofol and traditional anesthesia for Gastrointestinal endoscopy: a systematic review and meta-analysis. Clin Gastroenterol Hepatol 15(2):194–206 27451091 10.1016/j.cgh.2016.07.013

[10] Goyal R, Hasnain S, Mittal S, Shreevastava S (2016) A randomized, controlled trial to compare the efficacy and safety profile of a dexmedetomidine-ketamine combination with a propofol-fentanyl combination for ERCP. Gastrointest Endosc 83(5):928–933 26364968 10.1016/j.gie.2015.08.077

[11] Liu J, Liu X, Peng LP, Ji R, Liu C, Li YQ (2020) Efficacy and safety of intravenous Lidocaine in propofol-based sedation for ERCP procedures: a prospective, randomized, double-blinded, controlled trial. Gastrointest Endosc 92(2):293–300 32156544 10.1016/j.gie.2020.02.050

[12] Goudra B, Gouda G, Mohinder P (2020) Recent developments in drugs for GI endoscopy sedation. Dig Dis Sci 65(10):2781–2788 31916088 10.1007/s10620-020-06044-5

[13] Chen G, Mannens G, De Boeck M, Daly EJ, Canuso CM, Teuns G et al (2022) Comments to pharmacological and behavioral divergence of ketamine enantiomers by Jordi Bonaventura et al. Mol Psychiatry 27(4):1860–1862 35177823 10.1038/s41380-022-01447-4 PMC9126803

[14] Bonaventura J, Lam S, Carlton M, Boehm MA, Gomez JL, Solís O et al (2021) Pharmacological and behavioral divergence of ketamine enantiomers: implications for abuse liability. Mol Psychiatry 26(11):6704–6722 33859356 10.1038/s41380-021-01093-2 PMC8517038

[15] Pfenninger EG, Durieux ME, Himmelseher S (2002) Cognitive impairment after small-dose ketamine isomers in comparison to equianalgesic racemic ketamine in human volunteers. Anesthesiology 96(2):357–366 11818769 10.1097/00000542-200202000-00022

[16] Zhao L, Li Z, Jin B, Hou N, Yang H (2024) Safety and efficacy of low-dose Esketamine in laparoscopic cholecystectomy: a prospective, double-blind randomized controlled trial. BMC Anesthesiol 24(1):47 38302944 10.1186/s12871-024-02429-5 PMC10832235

[17] Gao L, Zhang Z, Zhu Y, Lu X, Tian Y, Wei L (2024) Effect of pretreatment with a small dose of Esketamine on sufentanil-induced cough during anesthesia induction: a randomized controlled trial. BMC Anesthesiol 24(1):116 38528479 10.1186/s12871-024-02501-0 PMC10964693

[18] Page MJ, McKenzie JE, Bossuyt PM, Boutron I, Hoffmann TC, Mulrow CD et al (2021) The PRISMA 2020 statement: an updated guideline for reporting systematic reviews. BMJ 372:n71 33782057 10.1136/bmj.n71 PMC8005924

[19] Wan X, Wang W, Liu J, Tong T (2014) Estimating the sample mean and standard deviation from the sample size, median, range and/or interquartile range. BMC Med Res Methodol 14:135 25524443 10.1186/1471-2288-14-135 PMC4383202

[20] Sterne JAC, Savović J, Page MJ, Elbers RG, Blencowe NS, Boutron I et al (2019) RoB 2: a revised tool for assessing risk of bias in randomised trials. BMJ 366:l4898 31462531 10.1136/bmj.l4898

[21] R Core Team (2024) R: A Language and environment for statistical computing. R Foundation for Statistical Computing, Vienna, Austria

[22] Posit Team (2024) RStudio: integrated development environment for R. Posit Software, PBC, Boston, MA

[23] Balduzzi S, Rücker G, Schwarzer G (2019) How to perform a meta-analysis with R: a practical tutorial. Evid Based Ment Health 22:153–160 31563865 10.1136/ebmental-2019-300117 PMC10231495

[24] Liu D, Gao X, Zhuo Y, Cheng W, Yang Y, Wu X et al (2025) Effect of Esketamine on cognitive recovery after Propofol sedation for outpatient colonoscopy: a randomized clinical trial. Drug Des Devel Ther 19:425–437 39867863 10.2147/DDDT.S503129 PMC11762454

[25] Xiao L, Zhang Z, Lu J, Liu Z, Zhang J, Kang L et al (2024) Efficacy and safety of Esketamine combined with Propofol for conscious sedation in painless colonoscopy: a prospective, randomized, double-blind controlled clinical trial. BMC Anesthesiol 24(1):394 39478485 10.1186/s12871-024-02779-0 PMC11523800

[26] Fu M, Sheng B, Liu R, Li Y, Chen G, Chen H et al (2024) Impact of different doses of esketamine on the incidence of hypotension in propofol-based sedation for colonoscopy: a randomized controlled trial. Ther Adv Drug Saf 15:20420986241278499 39314988 10.1177/20420986241278499 PMC11418320

[27] Ma Y, Wang J, Yang Y, Yao M (2024) Efficacy and safety of Esketamine combined with Propofol for curative endoscopic resection in colorectum: a prospective, randomized controlled trial. BMC Anesthesiol 24(1):96 38459471 10.1186/s12871-024-02475-z PMC10924399

[28] Sun X, Du Q, Liang Y, Tang L, Wei Q, Guo P et al (2025) Effect of low-dose Esketamine combined with Propofol on postoperative fatigue in colonoscopy: a randomized clinical trial. Ther Clin Risk Manag 21:807–816 40463767 10.2147/TCRM.S521961 PMC12129815

[29] Eberl S, Koers L, van Hooft J, de Jong E, Hermanides J, Hollmann MW et al (2020) The effectiveness of a low-dose esketamine versus an alfentanil adjunct to propofol sedation during endoscopic retrograde cholangiopancreatography: a randomised controlled multicentre trial. Eur J Anaesthesiol 37(5):394–401 31860599 10.1097/EJA.0000000000001134

[30] Guan Y, Pan H, Cong X, Fang F, Du S, Wang X et al (2024) Effect of esketamine on haemodynamic fluctuations in patients undergoing hysteroscopic surgery: a prospective, double-blind randomized clinical trial. Br J Clin Pharmacol 90(11):2754–2762 38958172 10.1111/bcp.16165

[31] Jonkman K, Van Rijnsoever E, Olofsen E, Aarts L, Sarton E, Van Velzen M et al (2018) Esketamine counters opioid-induced respiratory depression. Br J Anaesth 120(5):1117–1127 29661389 10.1016/j.bja.2018.02.021

[32] Sigtermans M, Dahan A, Mooren R, Bauer M, Kest B, Sarton E et al (2009) S(+)-ketamine effect on experimental pain and cardiac output: a population pharmacokinetic-pharmacodynamic modeling study in healthy volunteers. Anesthesiology 111(4):892–903 19741495 10.1097/ALN.0b013e3181b437b1

[33] Daly EJ, Singh JB, Fedgchin M, Cooper K, Lim P, Shelton RC et al (2018) Efficacy and safety of intranasal Esketamine adjunctive to oral antidepressant therapy in treatment-resistant depression: a randomized clinical trial. JAMA Psychiatr 75(2):139–148 10.1001/jamapsychiatry.2017.3739 PMC5838571 29282469

[34] Srinivasan SS, Alshareef A, Hwang A, Byrne C, Kuosmanen J, Ishida K et al (2023) A vibrating ingestible bioelectronic stimulator modulates gastric stretch receptors for illusory satiety. Sci Adv 9(51):eadj3003 38134286 10.1126/sciadv.adj3003 PMC10745699

[35] Liu X, Xiao Q, Zhuang S (2023) Comparison of propofol-esketamine versus propofol for anesthesia in gastroscopy: a double-blind, randomized controlled clinical trial. Front Med 10:1184709 10.3389/fmed.2023.1184709 PMC10442552 37614948

[36] Hori K, Nagasaka H (2002) Effects of Fentanyl on cardiovascular and plasma catecholamine responses in surgical patients. J Anesth 16(3):187–193 14517638 10.1007/s005400200022

[37] Deng J, Yu YF, Tang ZG, Lei HJ, Tan CC (2024) Efficacy and safety of low-dose Esketamine for painless gastrointestinal endoscopy in adults: a systematic evaluation and meta-analysis. Front Pharmacol 15:1364546 38645560 10.3389/fphar.2024.1364546 PMC11026590

[38] Zhou S, Zhu Z, Dai W, Qi S, Tian W, Zhang Y et al (2021) National survey on sedation for gastrointestinal endoscopy in 2758 Chinese hospitals. Br J Anaesth 127(1):56–64 33685636 10.1016/j.bja.2021.01.028

[39] Song N, Yang Y, Zheng Z, Shi WC, Tan AP, Shan XS et al (2023) Effect of esketamine added to propofol sedation on desaturation and hypotension in bidirectional endoscopy: a randomized clinical trial. JAMA Netw Open 6(12):e2347886 38117498 10.1001/jamanetworkopen.2023.47886 PMC10733809

[40] Chiang MH, Wu SC, You CH, Wu KL, Chiu YC, Ma CW et al (2013) Target-controlled infusion vs. manually controlled infusion of propofol with alfentanil for bidirectional endoscopy: a randomized controlled trial. Endoscopy 45(11):907–914 24165817 10.1055/s-0033-1344645

[41] De Oliveira GS Jr, Fitzgerald PC, Hansen N, Ahmad S, McCarthy RJ (2014) The effect of ketamine on hypoventilation during deep sedation with midazolam and propofol: a randomised, double-blind, placebo-controlled trial. Eur J Anaesthesiol 31(12):654–662 10.1097/EJA.0000000000000025 24247410

[42] Aroni F, Iacovidou N, Dontas I, Pourzitaki C, Xanthos T (2009) Pharmacological aspects and potential new clinical applications of ketamine: reevaluation of an old drug. J Clin Pharmacol 49(8):957–964 19546251 10.1177/0091270009337941

[43] Lee T (1991) Pharmacology of propofol. Ann Acad Med Singap 20(1):61–65 2029166

[44] Short CE, Bufalari A (1999) Propofol anesthesia. Vet Clin North Am Small Anim Pract 29(3):747–778 10332821 10.1016/s0195-5616(99)50059-4

[45] Yang H, Zhao Q, Chen H, Liu W, Ding T, Yang B et al (2022) The median effective concentration of propofol with different doses of esketamine during gastrointestinal endoscopy in elderly patients: a randomized controlled trial. Br J Clin Pharmacol 88(3):1279–1287 34496448 10.1111/bcp.15072

