# Supplementary material for: Efficacy and safety of esketamine for sedation during colonoscopy: A systematic review and Meta-analysis of randomized controlled trials
Source: Eur J Clin Pharmacol. 2026 Jan 17;82(2):28. doi: 10.1007/s00228-025-03935-2 (PMC12812101; doi:10.1007/s00228-025-03935-2)
Supplement: Supplementary file 1 — Supplementary Material 1 (25.5 KB) [file 228_2025_3935_MOESM1_ESM.docx]

**Title:**

**Efficacy and Safety of Esketamine for Sedation during Colonoscopy: A Systematic Review and Meta-analysis of Randomized Controlled Trials.**

**Running Title:**

Esketamine Sedation In Colonoscopy.

**Authors:**

Zainab Hussein^1^, Amira Mohamed Taha^2^, Alaa Abdrabou Abouelmagd^3^, Mohamed Nasser Elshabrawi^4^, Abdul Karim Durvesh^5^, Eman Ayman Nada^6,7^, Mohamed Abuelazm^8^, Mohamed Elnaggar^8^, Ismail Elkhattib^9^

**Affiliations.**

1. Faculty of Medicine, Minia Univerity, Minia, Egypt
2. Faculty of Medicine,Fayoum University, Fayoum, Egypt
3. Faculty of Medicine, South Valley University, Qena, Egypt
4. Clinical research department,Aswan heart center, Magdi Yaqoup Foundation, Aswan , Egypt
5. Sindh Institute of Advanced Endoscopy and Gastroenterology (SIAG), Karachi, Pakistan
6. Faculty of pharmacy, Tanta university, Gharbia, Egypt
7. Ministry of health, Damietta, Egypt
8. Faculty of Medicine, Tanta University, Tanta, Egypt.
9. Hartford Healthcare, Hartford, CT, USA
10. University of Nebraska Medical Centre, Omaha, NE, USA

**Keywords:**

Endoscopy; propofol; delirium; anaesthesia; endoscope.

**Contents:**

**Tables.**Table S1: Search strategy.

Table S2: Sensitivity analysis.

| Database | Search Terms | Search Results |
| --- | --- | --- |
| Pubmed | 1. **("Colonoscopy"[Mesh]** OR "colonoscop*"[Title/Abstract] OR "colonic endoscop*"[Title/Abstract] OR "lower GI endoscop*"[Title/Abstract] OR "lower endoscop*"[Title/Abstract] OR "sigmoidoscop*"[Title/Abstract] OR "proctosigmoidoscop*"[Title/Abstract] OR "colonoendoscop*"[Title/Abstract] OR ("colon"[Title/Abstract] AND ("endoscop*"[Title/Abstract] OR "scop*"[Title/Abstract])) OR "large bowel endoscop*"[Title/Abstract] OR "colorectal cancer screening"[Title/Abstract]) 2. **("Esketamine"[Title/Abstract]** OR "esketamine"[Title/Abstract] OR "S-ketamine"[Title/Abstract] OR "S ketamine"[Title/Abstract] OR "Spravato"[Title/Abstract] OR "JNJ-54135419"[Title/Abstract]) 3. **#1 AND #2** | 13 |
| Cochrane | 1. **("Esketamine" OR "esketamine" OR "S-ketamine" OR "S ketamine" OR "Spravato" OR "JNJ-54135419")** in Title Abstract Keyword 2. **(("Colonoscopy" OR "colonoscop*" OR "colonic endoscop*" OR "lower GI endoscop*" OR "lower endoscop*" OR "sigmoidoscop*" OR "proctosigmoidoscop*" OR "colonoendoscop*" OR "colon") AND ("endoscop*" OR "scop*" OR "large bowel endoscop*" OR "colorectal cancer screening"))** in Title Abstract Keyword 3. **#1 AND #2** | 6 |
| WOS | **TS=("Esketamine" OR "esketamine" OR "S-ketamine" OR "S ketamine" OR "Spravato" OR "JNJ-54135419")** 2. **TS=(("Colonoscopy" OR "colonoscop*" OR "colonic endoscop*" OR "lower GI endoscop*" OR "lower endoscop*" OR "sigmoidoscop*" OR "proctosigmoidoscop*" OR "colonoendoscop*" OR "colon") AND ("endoscop*" OR "scop*" OR "large bowel endoscop*" OR "colorectal cancer screening"))** 3. **#1 AND #2** | 16 |
| SCOPUS | **( TITLE-ABS-KEY ( "Esketamine" )** OR TITLE-ABS-KEY ( "esketamine" ) OR TITLE-ABS-KEY ( "S-ketamine" ) OR TITLE-ABS-KEY ( "S ketamine" ) OR TITLE-ABS-KEY ( "Spravato" ) OR TITLE-ABS-KEY ( "JNJ-54135419" ) ) **AND** **( TITLE-ABS-KEY ( "Colonoscopy" )** OR TITLE-ABS-KEY ( "colonoscop*" ) OR TITLE-ABS-KEY ( "colonic endoscop*" ) OR TITLE-ABS-KEY ( "lower GI endoscop*" ) OR TITLE-ABS-KEY ( "lower endoscop*" ) OR TITLE-ABS-KEY ( "sigmoidoscop*" ) OR TITLE-ABS-KEY ( "proctosigmoidoscop*" ) OR TITLE-ABS-KEY ( "colonoendoscop*" ) OR ( TITLE-ABS-KEY ( "colon" ) AND ( TITLE-ABS-KEY ( "endoscop*" ) OR TITLE-ABS-KEY ( "scop*" ) ) ) OR TITLE-ABS-KEY ( "large bowel endoscop*" ) OR TITLE-ABS-KEY ( "colorectal cancer screening" ) ) | 26 |

*Table S1: Search Strategy.*

| ***Outcome & Analysis Model*** | ***Study omitted*** | ***Esketamine (n)*** | ***Control (n)*** | ***No. of trials*** | ***Pooled MD/RR*** | ***95% CI*** | ***p-value*** | ***τ²*** | ***I² (%)*** |
| --- | --- | --- | --- | --- | --- | --- | --- | --- | --- |
| *Hypotension  (Random-effects model, RR)* | *All studies* | *347* | *295* | *4* | *0.34* | *[0.22, 0.53]* | *<0.0001* | *0.1119* | *58* |
|  | *Omitting Liu et al. 2025* | *221* | *169* | *3* | *0.3* | *[0.15, 0.61]* | *0.0008* | *0.2608* | *72* |
|  | *Omitting Fu et al. 2024* | *272* | *270* | *3* | *0.39* | *[0.25, 0.62]* | *<0.0001* | *0.077* | *55.2* |
|  | *Omitting Ma et al. 2024* | *266* | *216* | *3* | *0.28* | *[0.17, 0.46]* | *<0.0001* | *0.0668* | *35.9* |
|  | *Omitting Xiao et al. 2024* | *282* | *230* | *3* | *0.39* | *[0.27, 0.58]* | *<0.0001* | *0.0588* | *50.1* |
| *Injection Pain  (Random-effects model, RR)* | *All studies* | *267* | *266* | *3* | *0.42* | *[0.19, 0.97]* | *0.042* | *0.4468* | *80.5* |
|  | *Omitting Liu et al. 2025* | *141* | *140* | *2* | *0.42* | *[0.09, 1.89]* | *0.2565* | *1.065* | *90* |
|  | *Omitting Sun et al. 2025* | *191* | *191* | *2* | *0.31* | *[0.14, 0.68]* | *0.0036* | *0.2634* | *79* |
|  | *Omitting Xiao et al. 2024* | *202* | *201* | *2* | *0.59* | *[0.30, 1.19]* | *0.142* | *0.1748* | *67.1* |
| *Injection Pain  (Random-effects model, RR)* | *All studies* | *282* | *230* | *3* | *-0.23* | *[-0.50, 0.04]* | *0.0892* | *0.0276* | *50.7* |
|  | *Omitting Fu et al. 2024* | *207* | *205* | *2* | *-0.34* | *[-0.53, -0.19]* | *0.0007* | *0* | *0* |
|  | *Omitting Liu et al. 2025* | *156* | *104* | *2* | *-0.15* | *[-0.69, 0.39]* | *0.5878* | *0.1137* | *74.2* |
|  | *Omitting Ma et al. 2024* | *201* | *151* | *2* | *-0.11* | *[-0.55, 0.32]* | *0.6059* | *0.0669* | *65.8* |

*Table S2: Sensitivity analysis.*
